# Group A *Streptococcus* Disease Outbreak Associated with Large Congregate Shelter, Chicago, Illinois, USA, October 2023–January 2024

**DOI:** 10.3201/eid3206.250726

**Published:** 2026-06

**Authors:** Karrie-Ann Toews, Lauren Tietje, Anne Meyers, Marco Ciaccio, Stephanie Gretsch, Alyse Kittner, Kendall Anderson, Matthew Bertagna, Elizabeth S. Davis, Stockton Mayer, Rebecca M. Singer, Margaret Mary Butler, Diana Arce-Garza, Thomas D. Huggett, Ryan Fabrizius, Jennifer Levy, Stephanie Black, Sopio Chochua, Christopher J. Gregory, Michelle Funk

**Affiliations:** Chicago Department of Public Health, Chicago, Illinois, USA (K.-A. Toews, L. Tietje, A. Meyers, M. Ciaccio, S. Gretsch, A. Kittner, K. Anderson, M. Bertagna, J. Levy, S. Black, M. Funk); Centers for Disease Control and Prevention, Atlanta, Georgia, USA (K.-A. Toews, S. Chochua, C.J. Gregory); Rush University Medical Center, Chicago (E.S. Davis, M.M. Butler); University of Illinois Chicago, Chicago (S. Mayer, R.M. Singer, D. Arce-Garza); Lawndale Christian Health Center, Chicago (T.D. Huggett, R. Fabrizius)

**Keywords:** bacteria, Streptococcus pyogenes, group A streptococcus, congregate shelter, Chicago, Illinois, United States

## Abstract

Chicago Department of Public Health identified 3 pediatric patients hospitalized with group A *Streptococcus* (GAS) disease during October 26–November 3, 2023, in a large congregate family migrant shelter in Chicago, Illinois, USA. One patient had invasive GAS infection; 2 had peritonsillar abscesses requiring drainage. Despite infection control measures, GAS pharyngitis cases continued through November 13. Chicago Department of Public Health coordinated clinical partners to perform rapid antigen detection testing and throat cultures for residents and staff with pharyngitis symptoms. During November 20, 2023–January 3, 2024, a total of 428 symptomatic persons were evaluated; 166 tested positive for GAS. Among persons with GAS pharyngitis, median age was 12 years (range 0–45 years); 54.2% were women and girls. Common symptoms included sore throat (87.3%) and fever (63.3%). One pediatric resident death caused by invasive GAS was confirmed postmortem. This response highlights outbreak challenges in large congregate shelters housing children.

From late 2022 through 2023, multiple countries marked increases in group A *Streptococcus* (GAS) infections, including less severe illnesses and invasive infections, especially in children <10 years of age (*1*,*2*). Invasive GAS (iGAS) infections increased in 10 US states during 2013–2022 and surged in the United States and Europe during 2022–2023 (*3*,*4*). GAS colonization is more common in children, in whom asymptomatic throat carriage ranges from 5% to 15% (*5*). Persons experiencing homelessness (PEH) have a higher risk for iGAS disease compared with the general population (*6*,*7*). Outbreaks of invasive and noninvasive GAS infections in PEH have been described predominantly in adults and have been linked to alcohol or injection drug use and wounds or other skin breakdown (*7*–*10*). Clusters of GAS infections in shelter settings have been characterized by whole-genome sequencing and have required rapid response activities to mitigate transmission (*8*,*10*). However, the transmission of GAS infections within homeless shelters is poorly understood, particularly in shelters that house children.

During August 31, 2022, through December 31, 2024, more than 50,000 migrant new arrivals, predominantly from Venezuela, traveled to Chicago, Illinois, USA, and the city of Chicago opened 27 shelters to house them (*11*). Shelters housed either families with children <18 years of age or single adults; for single adults, men and women were housed in sex-specific areas of the same building. Shelters ranged from hotel-based facilities with 1–2 families per room to warehouses with large open rooms housing hundreds of persons.

We describe the public health response to a GAS disease outbreak in a large, congregate new arrival shelter (shelter A) with a rapidly expanding census of ≈1,300–1,700 residents during October 26, 2023–January 3, 2024. Approximately 50% of the population housed at shelter A was <18 years of age.

## Methods

iGAS infections are a reportable condition in Illinois and are defined as isolation of GAS from a normally sterile body site (e.g., blood or cerebrospinal fluid) or from a wound culture accompanied by necrotizing fasciitis or streptococcal toxic shock syndrome. Chicago Department of Public Health (CDPH) investigates all laboratory and provider-reported cases of iGAS disease in the city of Chicago. Investigations include identifying the clinical manifestations of infection, evaluating potential risk factors, and providing prevention and control guidance for cases that occur in a congregate or healthcare setting. Noninvasive GAS infections are those with clinically compatible illness and detection of GAS from a nonsterile site (e.g., throat or wound) and are not reportable in Illinois. The Illinois Department of Public Health defines an outbreak of GAS disease as either >2 epidemiologically linked iGAS disease cases or >1 iGAS disease case and >1 noninvasive GAS disease case that are epidemiologically linked within a 4-month period. We defined suspected cases in this outbreak as illness in persons at shelter A with symptoms consistent with pharyngitis that did not involve laboratory testing.

CDPH provides guidance and technical support to shelter facilities in infection prevention and control, outbreak response, disease reporting, behavioral health, maternal neonatal and child health, and healthcare navigation, and collaborates with healthcare providers serving this population. During August 2022–December 2024, healthcare for immigrant populations was provided predominantly by a health clinic associated with a large, publicly funded healthcare system. CDPH also funds shelter-based service teams that provide onsite clinical care at the shelters.

During the week beginning November 6, 2023, three cases of GAS infection requiring hospitalization were reported in residents of shelter A, who had symptom onset dates ranging from October 23 to November 3. One case was invasive, and 2 cases required surgical drainage of peritonsillar abscesses. All 3 cases were in children (<18 years of age); 2 cases were in children <5 years of age. Shelter A opened to house new arrivals approximately 3 weeks before the identification of the initial case. At that time, living quarters within the shelter were 3 large open congregate rooms on the first floor with multiple shared bathrooms and 2 large congregate eating spaces. Shelter A’s capacity expanded from 3 large rooms for congregate living in November 8, 2023, to 8 congregate rooms in January 3, 2024 (housing ≈1,300–1,700 persons total). Data on admissions to and exits from the shelter are unavailable because admissions and exits during that period were fluid. CDPH conducted a site visit at shelter A on November 7 to identify spaces for isolation, introduce provider reporting, and implement an infection prevention assessment tool, which collected information on resident population, infection control practices, environmental cleaning, clinical partners, protocols for resident illness, isolation and exclusion policies, and disease reporting. On November 13, a CDPH-funded primary care team visited the shelter and evaluated ill persons. During November 3–13, CDPH identified additional residents of shelter A with suspected GAS pharyngitis. Beginning November 20, CDPH implemented enhanced case-finding and treatment for residents and staff.

Because resources for facilitywide screening and treatment were not available, CDPH collaborated with clinical partners to assess symptomatic residents and staff, conduct GAS testing, and provide treatment. Before the testing event, shelter managers distributed CDPH-developed Spanish-language handouts regarding GAS pharyngitis symptoms and the importance of testing and treatment, so that persons with sore throat or other compatible symptoms would seek care. During testing events, shelter managers and contracted clinical partners visited each room in shelter A to recruit residents for screening of symptoms consistent with GAS pharyngitis and refer symptomatic residents for onsite testing (*12*). Providers collected demographic information, sleeping location within the shelter, and symptoms; assessed symptomatic persons for signs of clinical instability; and, if needed, arranged emergency medical service transport. If patients were clinically stable, providers evaluated patients and performed a streptococcal rapid antigen detection test (RADT) to enable immediate diagnosis and treatment and collected a second swab specimen for culture. Because antimicrobial susceptibility testing results for 1 of the initial 3 hospitalized case-patients was pansusceptible, we administered azithromycin to persons testing positive on RADT (a regimen based on Centers for Disease Control and Prevention [CDC] guidance (*13*) and selected because of its short course and availability of liquid formulation for pediatric dosing), and we advised these patients to wear a face mask. Persons subsequently testing positive by culture received antibiotics if they could be located after culture results were received. We recommended that persons testing negative on RADT receive additional clinical evaluation. If clinical suspicion of streptococcal pharyngitis remained high, especially in the context of close contacts testing positive on RADT, we administered antibiotics empirically. The CDC National Center for Immunization and Respiratory Diseases Division of Bacterial Diseases Streptococcus Laboratory and CDPH’s locally contracted laboratory provided culture results. Provider teams visited shelter A for testing and treatment 3 times weekly for the first week and then decreased to once per week as resources allowed. The last testing and treatment event occurred on January 3, 2024, because the number of symptomatic persons seeking screening and the percentage testing positive declined and resources were prioritized to address an active varicella outbreak among residents of shelter A.

We sent bacterial cultures from the first testing event to the CDC Streptococcus Laboratory for strain characterization. The laboratory performed whole-genome sequencing (WGS) and single-nucleotide polymorphism (SNP) analyses to determine *emm* types and antimicrobial susceptibility and to verify temporal relatedness of the isolates. A commercial laboratory performed subsequent bacterial cultures, which were not available for WGS. We used results from WGS for surveillance purposes only and did not use them for diagnosis, treatment, assessment of patient health, or case management.

CDPH’s activities were reviewed by the CDPH Institutional Review Board chair. The activities were determined to not constitute research and to be exempt from internal review board review.

## Results

### Surveillance, Outbreak Detection, Outbreak Response

**Figure 1:**
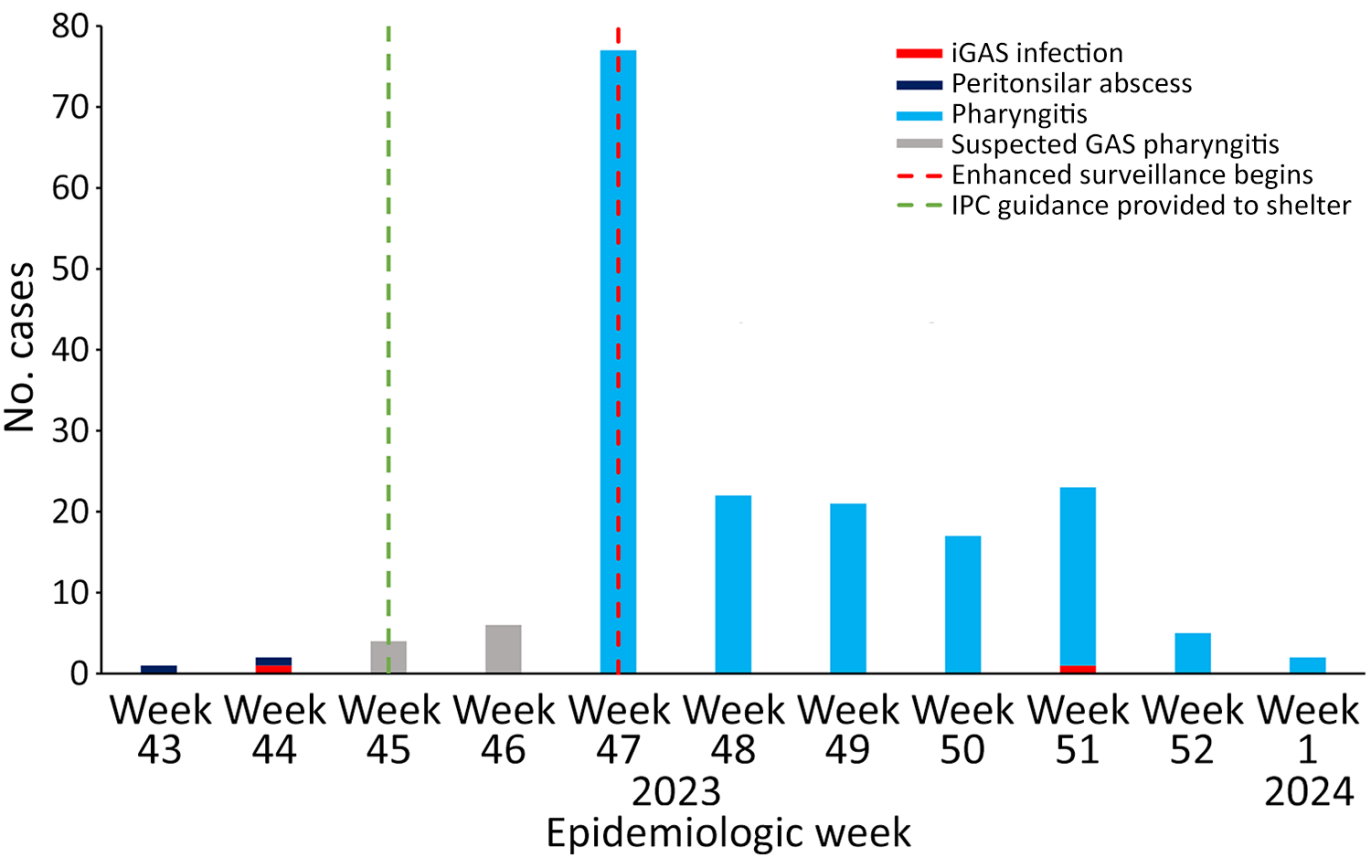
Epidemiologic curve for GAS *cases* in shelter A, by year and epidemiologic week, Chicago, Illinois, USA, October 25, 2023–January 3, 2024. Vertical dashed lines indicate key response activities. Suspect cases were identified clinically, without rapid or laboratory testing. Suspect cases identified clinically without rapid or laboratory testing. GAS, group A *Streptococcu*s; iGAS, invasive GAS; IPC, infection prevention and control.

During November 20, 2023–January 3, 2024, we tested a total of 428 persons (425 residents and 3 staff) for GAS at shelter A. Among persons tested, 260 (60.7%) tested negative, 166 (38.8%) tested positive, and 2 (0.5%) had missing test results (Figure 1). Of those who tested positive, we identified 114 (68.7%) through RADT and 52 (31.3%) through culture only (Table). We screened 25 symptomatic persons during >1 testing event, 2 (8%) of whom tested positive on subsequent testing. We identified cases among persons in all 8 rooms of the shelter, but we found most cases (109 [65.1%]) among persons in 2 rooms where the initial case-patients requiring hospitalization were housed (rooms A and B). Persons tested positive persons were predominantly women and girls (90 [54.2%]). Median age of persons testing positive was 12 years (range 0–45 years); 93 (56.0%) persons were in the 0–17-year age category. One child who previously tested negative during screening had disseminated iGAS diagnosed by a medical examiner outside of CDPH testing efforts.

**Table:** Characteristics of 426 persons screened for *group A Streptococcus* during point prevalence survey screening in large congregate shelter, Chicago, Illinois, USA, November 20, 2023–January 3, 2024*

Characteristic	Identified through RADT, no. (%)	Identified through culture, no. (%)	Negative, no. (%)
Total	114 (100)	52 (100)	260 (100)
Resident	114 (100)	51 (98.1)	258 (99.2)
Staff	0 (0)	1 (1.9)	2 (0.8)
Sex			
M	54 (47.4)	21 (40.4)	108 (41.5)
F	60 (52.6)	30 (57.7)	152 (58.5)
Not reported	0	1 (1.9)	0
Age group, y			
0–17	61 (53.5)	32 (61.5)	135 (51.9)
18–29	27 (23.7)	14 (26.9)	57 (21.9)
30–39	22 (19.3)	6 (11.5)	52 (20.0)
40–49	4 (3.5)	0	13 (5.0)
50–59	0	0	2 (0.8)
≥60	0	0	1 (0.4)
Unknown	0	0	0
Room			
A	42 (36.8)	21 (40.4)	87 (33.5)
B	31 (27.2)	14 (26.9)	81 (31.2)
C	3 (2.6)	3 (5.8)	6 (2.3)
D	10 (8.8)	5 (9.6)	26 (10.0)
E	13 (11.4)	3 (5.8)	25 (9.6)
F	5 (4.4)	0	7 (2.7)
G	2 (1.8)	0	7 (2.7)
H	1 (0.9)	3 (5.8)	1 (0.4)
Unknown or unverified	7 (6.1)	1 (1.9)	18 (6.9)
NA	0	2 (3.8)	2 (0.8)
Symptoms			
Fever	76 (66.7)	29 (55.8)	162 (62.3)
Sore throat	102 (89.5)	43 (82.7)	212 (81.5)
Headache	72 (63.2)	26 (50.0)	146 (56.2)
Nausea	29 (25.4)	17 (32.7)	93 (35.8)
Vomiting	21 (18.4)	11 (21.2)	55 (21.2)
Abdominal pain	25 (21.9)	17 (32.7)	71 (27.3)
Difficulty breathing	35 (30.7)	21 (40.4)	108 (41.5)
Antibiotics prescribed	112 (98.2)	32 (61.5)	73 (28.1)

*Two cases had indeterminate results and are not included in this table. NA, not available; RADT, rapid antigen detection test.

We prescribed antibiotics to 144 (86.7%) persons testing positive and 73 (28.1%) symptomatic close contacts testing negative. Among persons testing positive through RADT, we treated 112 (98.2%) with antibiotics. Of the 52 who tested positive only through culture, 32 (61.5%) could be located and treated with azithromycin. We did not consider use of intramuscular benzathine G plus rifampin as a single dose because of limits on resources and operational capacity.

**Figure 2:**
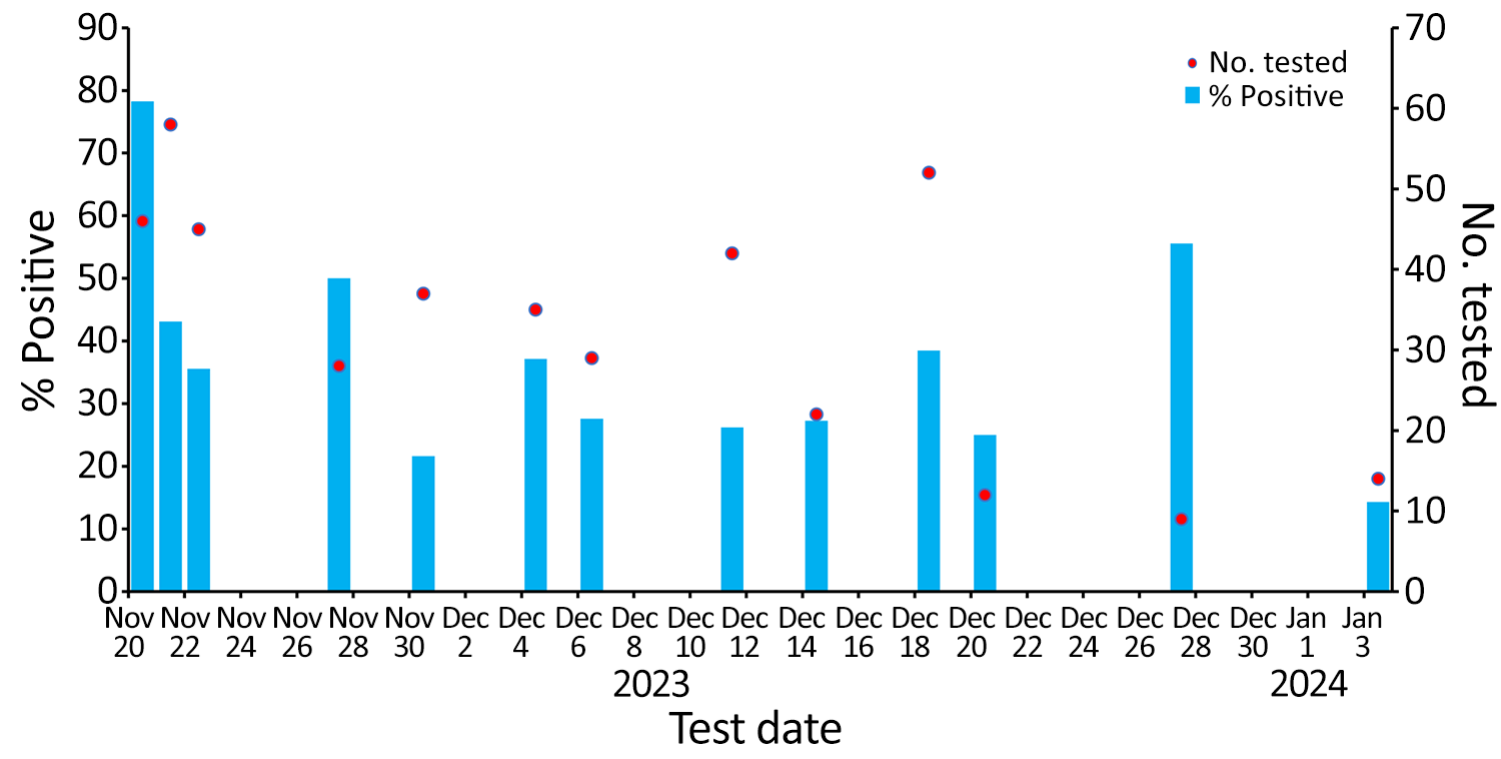
Group A *Streptococcus* percentage positivity on point prevalence screening dates and number of persons tested per day in shelter A, Chicago Illinois, USA, November 20, 2023–January 3, 2024.

During the outbreak, we conducted screening 13 times. On each testing day, 9–58 persons were tested (mean 33 persons). Screening positivity peaked on November 20, 2023, when 36 (78.3%) of 46 persons screened tested positive. The second-highest screening positivity occurred on December 27, 2023, when 5 (56.0%) of 9 persons screened tested positive (Figure 2). Because of rapid turnover, CDPH had no reliable mechanism to determine daily shelter census. We restricted GAS screening to those with clinically compatible symptoms. Close contacts of clinically compatible persons also were subject to screening at the discretion of the treating clinician. We observed no identifiable pattern to positivity during the screening period, and the number of persons screened varied because of the capacity of the testing teams. 

### Infection Control Assessment

After the November 5 infection control assessment, CDPH recommended that shelter A managers add signage for residents promoting hand hygiene and cough etiquette. Other recommendations were to ensure daily cleaning of bathrooms, coordinate transport of residents with respiratory illness to a contracted healthcare provider, retain onsite rapid COVID-19 and influenza testing capacity, and implement disease reporting.

### Laboratory Testing

Of 48 cultures sent for typing, 14 (29.2%) were *emm*1.0, 19 (39.6%) were *emm*12, and 15 (31.3%) were *emm*75. We detected all 3 *emm* types in rooms A and B. In total, sequencing identified 6 different GAS sublineages (2 *emm*1, 3 *emm*12, and 1 *emm*75). WGS analysis identified 2 different clusters of *emm*1 isolates; cluster 1 consisted of 5 isolates with 0–2 SNP differences, and cluster 2 consisted of 8 isolates with 0–7 SNP differences. Two isolates available from the initial 3 case-patients were *emm*1 that were genetically indistinguishable and pan-susceptible.

### Clinical Characteristics

Common symptoms among those persons in whom GAS pharyngitis was diagnosed were sore throat (87.3%), fever (63.3%), and headache (59.0%). Among persons testing negative on RADT, we noted similar commonly reported symptoms, including sore throat (81.5%), fever (62.3%), and headache (56.2%). Among iGAS case-patients, the person with the invasive case during the initial cluster had otitis interna and infected mastoid air cells. The other case-patient with invasive disease was deceased on arrival to the emergency department, and testing demonstrated COVID-19 and adenovirus co-infection postmortem. The decedent child had GAS isolated from blood, cerebrospinal fluid, liver, and spleen; cause of death was determined by the medical examiner to be GAS sepsis. No additional peritonsillar abscesses or hospitalizations attributable to GAS after CDPH’s response were reported.

## Discussion

We describe a GAS disease outbreak in a new-arrival shelter in Chicago. Unlike outbreaks in traditional homeless shelters, which generally house an older population with wounds serving as a key risk factor (*5*,*6*,*8*,*9*), new arrival shelters may have a younger population with fewer underlying conditions, and clinical risk factors for infection may include co-infection with routine pediatric illnesses. New arrival shelter populations in Chicago were more dynamic than the traditional homeless shelter population because census spikes and housing demand were tied closely to the volume of new arrivals, and such populations may require tailored communication approaches because of language differences.

In more well-documented settings like healthcare facilities, outbreaks of GAS disease often are approached aggressively with testing and treatment of all potentially exposed persons (*14*,*15*). In response to another GAS disease outbreak among adults experiencing homelessness in Alaska, CDC’s Arctic Investigations Program distributed treatment at sites frequently used by the population (e.g., shelters, soup kitchens, and supportive housing units) (*8*). The large and transient population in shelter A made neither of those approaches feasible. At the time of the outbreak, shelter A’s census increased by ≈400 residents within a month’s time, and lack of admission and exit tracking contributed to the challenge of quantifying this dynamic population. That rapid growth, coupled with limited public health resources, impeded deployment of shelterwide testing and treatment and necessitated targeted interventions. Because facilitywide screening or treatment was not feasible, we conducted testing of symptomatic persons at shelter A, aiming resources at persons more likely to spread infection among the shelter population.

Quick access to testing and treatment (including treatment of persons testing negative who had GAS-positive close contacts) onsite probably reduced the magnitude of spread. Because available laboratory data showed pansusceptibility, per CDC’s guidelines on antibiotic regimens for carriage eradication in a long-term care facility (*13*), we selected azithromycin because of its shorter 5-day course and availability of pediatric liquid formulation for ease of administration. We used contracted clinical partners, experienced in working with transient populations, to enable rapid and repeated assessment and testing of shelter A residents during a nearly 2-month period. Use of streptococcal RADT, in conjunction with throat cultures, enabled immediate treatment of persons testing positive. Throat cultures were still critical diagnostic tools given that 12.4% of persons tested were found to be GAS culture positive while testing negative on RADT. Of note, persons testing GAS positive through culture alone were less likely to receive antibiotics because the large and rapidly changing population in shelter A made it challenging to relocate persons after their initial contact with the medical team. This situation led to >20 symptomatic persons who did not receive treatment and could potentially continue to spread disease to others in the shelter.

Despite intensive clinical assessment, we were not able to prevent a pediatric death caused by iGAS. The decedent child had been tested for GAS infection because of symptom onset (including fever, sore throat, headache, nausea, and vomiting) 13 days before their sudden death occurred and had tested negative for GAS by RADT and culture. We administered no antibiotics for this patient. Circulating viruses probably contributed to the child’s death, given that infection with COVID-19 and adenovirus also were identified postmortem. That tragic outcome highlights the need for reevaluation and retesting of persistently symptomatic persons after a negative GAS test result, which is supported by the identification of 2 pharyngitis cases among the 25 persons tested >1 time. In addition, we could not assess the role of a language difference on health communication with this population in the context of this response. During a subsequent measles outbreak in a Chicago new arrival shelter, CDPH used a similar response infrastructure but also contracted community health workers to provide vaccine messaging. Those CDPH-contracted community health workers operated as part of the response architecture to provide culturally proficient vaccine messaging and validate resident comprehension. Our efforts substantially decreased the likelihood of a large measles outbreak in Chicago, and we hypothesize that similar messaging strategies may have led more shelter A residents with symptoms to seek care at our testing events during the iGAS response (*16*).

Although only a portion of isolates were able to be *emm* typed, we identified 3 *emm* types, suggesting multiple introductions of GAS to the shelter population as shelter A quickly expanded capacity. Further, multiple highly related isolates suggest transmission within the shelter. Adoption of routine entry screening or periodic screening in shelters could improve future outbreak response, contingent on available resources. Of note, the number of buses transporting new arrivals to Chicago tripled in number during this timeframe, and 27 shelters were opened.

The close aggregation of transient populations promotes transmission of viral illness (*17*), and the subsequent breakdown of skin from rash or respiratory epithelium from upper respiratory infections increases the risk for bacterial superinfections (*18*). We identified 1 co-infection with GAS and varicella rash in shelter A in January 2024, outside of the response period. Vaccine-preventable viral co-infections such as COVID-19 and varicella in a potentially vaccine-naive population may be contributing factors to GAS disease severity, hospitalization, and death. Varicella is not on the childhood vaccination schedule for many countries in South America and is not included on the childhood immunization list for Venezuela (*19*), which accounted for 88% of new arrivals arriving to Chicago during this period. Varicella infections frequently have been identified in new arrival shelter populations (*20*), resulting in multiple shelters in Chicago experiencing outbreaks. Shelter A was one of the most affected, having a large varicella outbreak beginning in January 2023. As GAS disease declined, public health resources were shifted to address that response as immunization teams were mobilized to prevent additional illness.

This outbreak highlights challenges with sheltering a highly dynamic population in a large, congregate space with a rapid increase in primary care needs. Although considerable pressure existed to house large numbers of new arrivals at the time, early containment of ill persons in an isolation space might have reduced transmission, overall disease prevalence, and hospitalization. Challenges included a daily dynamic shelter census, limited healthcare personnel resources, and limited ability to quantify symptomatic persons in an emergency shelter space not compatible with mass prophylaxis. Our response underscores the importance of access to healthcare and infection control resources in congregate shelter settings. CDPH’s partnership with vulnerable population response teams with regular onsite symptom assessment, testing, and treatment in a culturally proficient manner, including the use of residents’ language of choice, was instrumental in enabling a swift, well-coordinated, and prolonged response. WGS results provide evidence of multiple related transmission events in shelter A, underscoring transmission dynamics and the need for infection control measures in a congregate setting. Our findings augment previous experience that suggests the need for repeated assessment of continually symptomatic persons and communication recommending that those persons return for reevaluation may be critical in circumstances where whole facility screening is not feasible.
